# Association Between Obstructive Sleep Apnea and Periodontitis: A Systematic Umbrella Review

**DOI:** 10.1002/cre2.70300

**Published:** 2026-04-26

**Authors:** Mohammed Khalid Mahmood, Shahen Hiwa Omer, Sarhang Sarwat Gul, Yad Mariwan Mohammed Amin, Herve Tassery, Delphine Tardivo, Romain Lan

**Affiliations:** ^1^ College of Dentistry Sulaymaniyah University Sulaymaniyah Iraq; ^2^ Department of Dentistry Komar University of Science and Technology Sulaymaniyah Iraq; ^3^ Medical Laboratory Department, College of Health and Medical Technology Sulaymaniyah Polytechnic University Sulaymaniyah Iraq; ^4^ Department of Periodontics, College of Dentistry University of Sulaymaniyah Sulaymaniyah Iraq; ^5^ Department of Dentistry Tishk International University Sulaymaniyah Iraq; ^6^ Dental School of Medicine, Conservative and Endodontic Department Aix‐Marseille University Marseille France; ^7^ Marseille Hospital APHM IHU‐MEPHI Institute Marseille France; ^8^ Faculty of Medical and Paramedical Sciences, French National Center of Scientific Research (CNRS), French Blood Establishment (EFS), Bio‐Cultural Anthropology, Law, Ethics and Health Laboratory (ADES) Aix‐Marseille University Marseille France; ^9^ Oral Surgery Department, Timone University Hospital, Marseille Provence‐Alpes‐Côte d'Azur France

**Keywords:** meta‐analysis, obstructive sleep apnea, periodontitis, sleep disorders, systematic review

## Abstract

**Aim:**

The aim of this study was to evaluate the association between obstructive sleep apnea (OSA) and periodontitis using an umbrella meta‐analysis (MA).

**Methods:**

PubMed, MEDLINE, Web of Science, Scopus, Embase, and Google Scholar databases were systematically searched before December 2025. MAs reporting odds ratios (OR) for the association between OSA and periodontitis were included. Methodological quality was assessed using AMSTAR‐2, and certainty of evidence was evaluated using GRADE. Random‐effects MA was performed. Egger's test and trim‐and‐fill analysis were used to assess the publication bias.

**Results:**

Seven MAs encompassing over 225,000 participants were included. A significant association was found between OSA and periodontitis (OR 1.96, 95% CI 1.68–2.29, *p* < 0.0001), with substantial heterogeneity (*I* ^2^ = 69.5%). After using trim‐and‐fill method to adjust for publication bias, the association strengthened (OR 2.24, 95% CI 1.92–2.60, *p* < 0.0001). A dose–response pattern emerged, with severe OSA showing higher odds (OR 2.25) compared to mild–moderate OSA (OR 1.82), though not statistically different (*p* = 0.44). The association remained consistent among different study qualities and sample sizes. The GRADE assessment rated the certainty of evidence of the main outcome as low due to heterogeneity and publication bias. Further, a high overlap ratio of 26.8% was observed among the included MAs.

**Conclusions:**

The results of this review suggest a significant association between OSA and periodontitis, with a greater association of severe OSA with periodontitis. Future studies should examine the impact of periodontal therapy on OSA severity and vice versa.

**PROSPERO registration:**

CRD420251241137

## 1 Introduction

Obstructive sleep apnea (OSA) and periodontitis are relatively common chronic diseases in adult populations. OSA is a condition that causes the upper airway to obstruct or close multiple times per hour while sleeping, resulting in intermittent episodes of low blood oxygen levels and intermittent systemic inflammation (Lembo et al. 2021). On the other hand, periodontitis is a chronic infection of the supporting structures of the teeth initiated and progressed by dysbiotic dental biofilm of subgingival in a susceptible host, leading to irreversible damage to tooth supporting structures and tooth loss (Mahmood et al. 2024; Qadir et al. 2025; Abdulkareem et al. 2023). OSA and periodontitis have several shared risk factors, such as obesity, smoking, and diabetes, and both conditions have a chronic inflammatory nature (Bianchi et al. 2024; Mohammed et al. 2022).

Several factors may contribute to the development and progression of OSA, such as any physical anatomy that narrows the airway, obesity, and aging. Additionally, it has been reported that sex is another important contributor, as men are 2–3 times more likely to have OSA (Chang et al. 2020). On the other hand, certain oral anatomical features contribute to the risk of OSA, such as a large tongue, a low‐hanging soft palate, enlarged tonsils, or specific jaw structures, all of which may lead to narrowing of the airway (Chan et al. 2010). Long‐term OSA increases the risk for hypertension and cardiovascular problems, diabetes mellitus, cognitive disorders, daytime dizziness, and a decrease in quality of life (Batool‐Anwar et al. 2016; Al Lawati et al. 2009). Moreover, OSA has shown increased risk of xerostomia, periodontitis, dental caries, and bruxism (Chan et al. 2010; Maniaci et al. 2024a).

There is a biological basis for the possible relationship between OSA and periodontitis. For example, mouth breathing caused by OSA results in reduced salivary flow and its antibacterial activities, thus creating a favorable environment for the development of periodontal pathogens (Schmidlin et al. 2020). Additionally, the intermittent hypoxemia associated with OSA triggers systemic inflammatory cytokines (C‐reactive protein, interleukin‐6, and tumor necrosis factor‐α), which can further exacerbate periodontal destruction. On the other hand, there is evidence that chronic periodontal inflammation can lead to upper airway inflammation, suggesting a reciprocal or bidirectional relationship (Lembo et al. 2021; Bianchi et al. 2023).

Although there has been increasing interest in studying the relationship between OSA and periodontitis, previous systematic reviews (SRs) and meta‐analyses (MAs) have reported a wide range of results, with ORs between 1.56 (Zhang et al. 2022) and 2.46 (Portelli et al. 2024). The variability in these studies' results is likely due to heterogeneity in the diagnostic criteria of each disease, variation in the demographic characteristics of the populations studied, and the degree of adjustment for confounders in the studies.

Therefore, this umbrella review aims to provide a synthesis of evidence from all available MAs that examined the association between OSA and periodontitis in adult populations. The purpose was to evaluate the pooled magnitude of the association, to evaluate if there is a dose–response relationship between the severity of one condition and the outcome of the other, to use the GRADE framework to evaluate the certainty of evidence, and to identify areas of future research to better inform clinical practice.

## 2 Materials and Methods

### 2.1 Protocol and Registration

The 2020 PRISMA standards were followed in conducting this umbrella review (Page et al. 2021), and relevant methodological recommendations for umbrella reviews (Fusar‐Poli and Radua 2018; Ioannidis 2009). The protocol for this study was prospectively registered under the registration number of (CRD420251241137) in the International Prospective Register of Systematic Reviews (PROSPERO).

### 2.2 Eligibility Criteria

The research questions of this review were formulated according to PECOS/T guideline as follows: (P) Problem: What is the pooled analysis of the association between OSA and periodontitis? Is there an increased risk of periodontal disease among patients with OSA? Is there a dose–response relation between periodontitis severity and OSA severity? Does periodontal therapy decrease the severity of OSA? (P) Population: Adult patients with OSA and periodontitis. (C) Comparison: Participants with a healthy periodontium and without OSA. (O) Outcome: Mostly OSA, but periodontitis in some studies. (S) Study design: All MAs that were published in the English language and have presented their pooled analysis in OR. (T) Time: MAs that were published in the mentioned databases before December 2025.

Since the included SRs had previously specified these categories when choosing their primary studies for inclusion, there was no need to define variables like OSA (and its subgroups) and periodontitis (and its subgroups). As a result, we simply gathered all of the variable categories together. All the included studies were observational, and no experimental studies were found to be included. However, whether the included reviews were cross‐sectional, case–control, or cohort studies, there were no limitations on the observational study design.

Reviews that examined one variable without examining the other, reported their findings in non‐comparable measurements, or did not use systematic procedures (narrative review, scoping review, etc.) were removed. To confirm the validity of the evidence found, conference abstracts, dissertations, editorials, and any other material not published in a peer‐reviewed journal were excluded as well.

### 2.3 Search Strategy

A combination vocabulary of MeSH terms and keywords associated with OSA, periodontitis, and SR, MA, a search method was developed. Table S1 contains the complete list of search phrases and combinations. Dates of publication were not restricted, and all the relevant records before December 2025 were targeted.

### 2.4 Study Selection

Duplicate records were eliminated after all of the records that were retrieved from databases were entered into the Zotero reference management tool. Two reviewers (M.M. and S.O.) independently carried out the study selection process in two stages. Part 1 involved screening abstracts and titles for possibly relevant studies based on eligibility criteria. Whereas Part 2 involved a detailed eligibility screening of potentially eligible full‐text articles. The MAs that matched all the predefined requirements were included. The two reviewers discussed and worked out any disagreements that arose during the screening process. A third reviewer (S.G.) was involved to obtain consensus.

### 2.5 Data Extraction

A structured Microsoft Excel spreadsheet made specifically for this review was used to extract data. Two reviewers (M.M. and S.O.) separately extracted the data in order to minimize variability and the possibility of errors. When the agreement could not be reached, a third reviewer (S.G.) was consulted in order to reconcile the conflicting data extraction.

In order to facilitate the systematic gathering of data by clinical factors and parameters, several spreadsheets were created within the Excel file for the main effect size in OR, OSA, and periodontitis categories, data stratified by study quality and sample size.

Key findings were extracted from the studies, like: first author's surname, year of publication, sample size, number of total participants, number of primary studies included in the quantitative analysis, measured variables, measurement tool of the exposure and outcomes, and the main results of each study. Since the main effect size of this review was OR, these were extracted for the pooled analysis.

### 2.6 Methodological Quality Assessment

The AMSTAR 2 (A Measurement instrument to Assess systematic Reviews) tool was used to assess the methodological quality of the included MAs (Shea et al. 2017). Each review is given a quality grade of high, moderate, poor, or severely low using the AMSTAR 2 tool. The seven domains that AMSTAR 2 deems “critical” are given particular attention. Each evaluation was completed independently by two reviewers (M.M. and S.O.), and disagreements were resolved by discussion or, if necessary, the participation of a third reviewer (S.G.).

### 2.7 Data Synthesis and Analysis

The random‐effects model developed by DerSimonian and Laird was employed to guarantee methodological consistency among the included outcomes (DerSimonian and Laird 1986). Since all the outcomes were in OR, these measures were obtained and reanalyzed.

The *I* ^2^ statistical test was used to evaluate study heterogeneity. For heterogeneity, a statistically significant *p* value was defined as less than 0.10. The Cochrane Handbook for Systematic Reviews of Interventions' recommendations for interpreting *I* ^2^ values were used as follows: values between 0% and 40% were deemed possibly inconsequential, moderate heterogeneity was indicated by 30%–60%, substantial heterogeneity by 50%–90%, and substantial heterogeneity by 75%–100% (Jpt 2008).

Additionally, we performed a Corrected Covered Area (CCA) analysis to find the overlap ratio between the included MAs. In addition, a statistical analysis was conducted to determine whether publication bias was present. To find small‐study effects, Egger's regression test was used; a *p* value of less than 0.05 suggested possible bias (Egger et al. 1997). Moreover, we conducted the trim‐and‐fill technique to compensate for the potential publication bias. Furthermore, we performed subgroup and sensitivity analyses based on OSA severity, study quality, and sample size. Cochrane's RevMan program, which can be accessed online (Fekete and Győrffy 2025), was used for all statistical analyses in this review.

### 2.8 Certainty of Evidence Assessment

The GRADE (Grading of Recommendations Assessment, Development and Evaluation) approach was used to assess the level of confidence in the evidence supporting the primary outcome of this umbrella review. GRADE framework evaluates the quality of evidence from various studies in a methodical and transparent manner, taking into account aspects such as methodological rigor, research design, consistency of findings, directness of evidence, imprecision, and risk of publication bias (Guyatt et al. 2008).

## 3 Results

### 3.1 Study Selection

**Figure 1:**
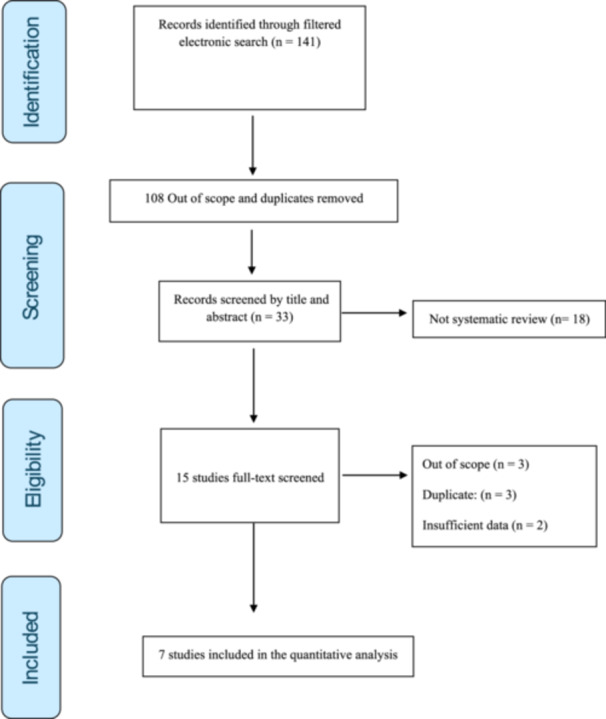
Study selection process.

The filtered search detected a total of 141 records. After deleting 108 out of scope and duplicates, 33 records remained for full‐text screening. Further, 18 non‐SR articles were excluded. Out of these 15 remaining papers, an additional eight records were excluded due to irrelevance to the research question (*n* = 3), were duplicates (*n* = 3), or had insufficient data (*n* = 2). Ultimately, seven unique MAs met the inclusion criteria (Zhang et al. 2022; Portelli et al. 2024; Al‐Jewair et al. 2015; Khodadadi et al. 2022; Liu et al. 2022; Molina et al. 2023; Zhu et al. 2023). Figure 1 illustrates the study selection procedure.

### 3.2 Characteristics of the Included SRs and MAs

Seven MAs were included in this umbrella review, collectively analyzing data from 23 unique primary studies encompassing more than 225,000 participants. The included studies were published from 2015 to 2024. All MAs focused exclusively on adult populations aged 18 years and older, with mean ages across studies typically ranging from 28 to 79 years. Periodontitis was assessed through various clinical parameters, including probing depth, clinical attachment loss, bleeding on probing, radiographic alveolar bone loss, and standardized classifications such as the CDC/AAP criteria. The OSA was diagnosed primarily through polysomnography using the apnea–hypopnea index, supplemented in some studies by validated questionnaires including the Berlin questionnaire, STOP‐BANG, and Epworth Sleepiness Scale (EPS). The reported ORs for the association between OSA and periodontitis ranged from 1.56 to 2.46, with most MAs providing additional stratified analyses by OSA severity, periodontitis severity, or adjusted for demographic confounders. Key characteristics of these included studies are presented in Table 1.

**Table 1:** Main characteristics of the included meta‐analyses.

Study	No. of included studies in MA	No. of cases	Age range (years)	Variables	Main findings
Al‐Jewair et al. (2015)	4	~30,130 participants across 6 studies	21–79 years (Adults)	− Clinical attachment loss (CAL) − Periodontal pocket depth (PPD) − Oral hygiene indices − Radiographic alveolar bone loss (ABL) − Salivary cytokines − OSA diagnosed by polysomnography or questionnaires	Pooled odds ratio = 1.65, 95%: 1.11, 2.46).
Khodadadi et al. (2022)	10	30,994 participants (8177 OSA‐positive, 22,817 OSA‐negative)	Mean age across studies: 28.5–55.9 years (Adults ≥ 18 years)	− Periodontitis (per 2017 or 1999 classification) − OSA diagnosed by PSG, AHI, questionnaires, or self‐report	Overall: OR = 2.17 (95% CI: 1.66–2.83) Adjusted for age/sex: OR = 1.75 (95% CI: 1.65–1.85) PSG: OR = 1.31 (95% CI: 1.20–1.43) Mild‐to‐moderate OSA: OR = 2.51 (95% CI: 1.32–4.78) Severe OSA: OR = 1.58 (95% CI: 0.70–3.58)
Molina et al. (2023)	6	47,024 participants	Adults ≥ 18 years Mean ages across studies ranged approximately 40–65 years	− Periodontitis (various definitions: CDC/AAP criteria, CAL loss, and PD) − OSA diagnosed by PSG, questionnaires (Berlin, STOP‐BANG), and AHI	OR = 1.65 (95% CI: 1.21–2.25)
Portelli et al. (2024)	10	88,040 participants	Adults (studies ranged from mean ages of approximately 28–65 years)	− Periodontitis (diagnosis based on clinical examination, PD, CAL, ABL, and BOP) − OSA diagnosed by PSG, classified by AHI	OR = 2.46 (95% CI: 1.73–3.49)
Zhang et al. (2022)	9	43,414 participants	Adults ≥ 18 years Mean ages across studies ranged approximately 39.5–61 years	− Periodontitis (diagnosed by clinical examination, including PD, CAL, BOP, PI, GI, REC, ABL, and calculus index; various definitions, including AAP/CDC criteria) − OSA is diagnosed primarily by PSG, ARES, or questionnaires (Berlin, ESS, STOP‐BANG)	OSA and risk of periodontitis (6 studies): OR = 1.56 (95% CI: 1.06–2.06).
Zhu et al. (2023)	10	31,800 participants	21–79 years; Mean ages across studies approximately 40–65 years (Adults ≥ 18 years)	− prevalence of periodontitis, CAL, PD, PI, GI, and percentage of BOP. − OSA	Prevalence of periodontitis: OR 2.348; 95% CI 2.22–2.48. PD: SMD = 0.681, 95% CI: 0.06–1.30, *p* = 0.031. CAL: SMD = 0.69, 95% CI: 0.16–1.22, *p* = 0.01.
Liu et al. (2022)	10	43,296 participants (11,773 SDB‐positive, 31,523 SDB‐negative)	Adults ≥ 18 years Mean ages across studies ranged approximately 29–56 years	− Periodontitis (diagnosed by clinical periodontal examination, using AAP/CDC definitions) − SDB diagnosed by PSG and AHI	Overall: OR = 1.83 (95% CI: 1.52–2.20) Severe periodontitis: OR = 1.39 (95% CI: 1.20–1.61) Mild SDB: OR = 1.66 (95% CI: 1.40–1.97) Moderate SDB: OR = 2.23 (95% CI: 1.22–4.08) Severe SDB: OR = 2.66 (95% CI: 1.54–4.58)

### 3.3 Quality of the SRs and MAs

The methodological quality of the included MAs was assessed using the AMSTAR‐2 tool, revealing that two reviews (28.6%) were rated as high quality, while five reviews (71.4%) were rated as moderate quality. Each of the seven reviews effectively addressed the elements of the PICO framework, utilized detailed search strategies for their literature searches, performed duplicate selections from included studies as well as duplicate extractions of study data, and appropriately applied statistical methodologies to perform MAs. The most common methodological limitation was that each review failed to register an a priori protocol or justify deviation(s) from said registered protocol (Questions 2 and 7), failed to identify/acknowledge funding source(s) for included studies (Question 10), and inadequately assessed the risk of bias upon individual study results (Question 12). Despite these limitations, all reviews satisfactorily addressed heterogeneity, investigated publication bias, and reported potential conflicts of interest, demonstrating overall adherence to SR reporting standards. Table S2 contains the full list of the evaluation of all domains for each study.

### 3.4 Overlap Analysis

Among the 7 MAs included, we identified 23 unique primary studies across 60 total study inclusions. The CCA was calculated at 26.81%, indicating very high overlap according to established thresholds (Pieper et al. 2014). Fifteen studies (65.2%) appeared in multiple MAs, with two studies (Keller et al. 2013; Seo et al. 2013) included in all seven reviews. Table S3 shows the summary of the overlap analysis.

### 3.5 Meta‐analysis Results

Overall, we conducted a total of five pooled analyses. Details of the findings are presented in Table 2.

**Table 2:** Meta‐analysis results of the association between obstructive sleep apnea and periodontitis.

Variables	No. of included estimates	OR (95% CI)	*p* Value	*I* ^2^ (95% CI), *p* heterogenicity	*p* Egger's test (Publication bias)	GRADE
Overall	7	1.96 (1.68, 2.29)	*p* < 0.0001	69.5%, *p* = 0.003	0.039	Low
Trim and fill	7	2.24 (1.92, 2.60)	*p* < 0.0001	73.7%, *p* < 0.0001		
OSA severity	2		Subgroup difference: *p* = 0.44			
Mild‐moderate OSA	2	1.82 (1.36, 2.43)		25.4%, *p* = 0.24		
Severe OSA	2	2.25 (1.40, 3.61)		6.4%, *p* = 0.30		
Study quality	7		Subgroup difference: *p* = 0.23			
High	2	1.78 (1.52, 2.09)		0%, *p* = 0.57		
Moderate	5	2.06 (1.72, 2.47)		61%, *p* = 0.03		
Sample size	7		Subgroup difference: *p* = 0.08			
Over 40,000	4	1.81 (1.52, 2.16)		36.2%, *p* = 0.19		
Under 40,000	3	2.21 (1.90, 2.57)		37.5%, *p* = 0.20		

**Figure 2:**
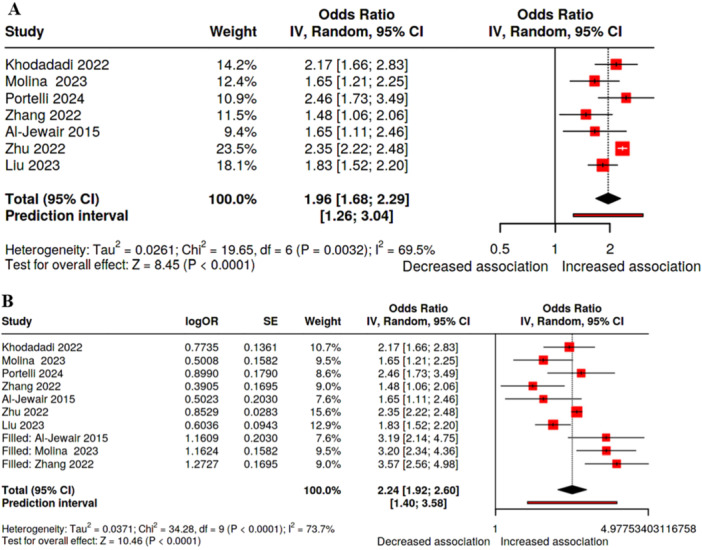
Forest plot of the association between obstructive sleep apnea and periodontitis. A. DerSimonian and Laird random‐effects model without trim and fill analysis. B. With trim‐and‐fill analysis.

**Figure 3:**
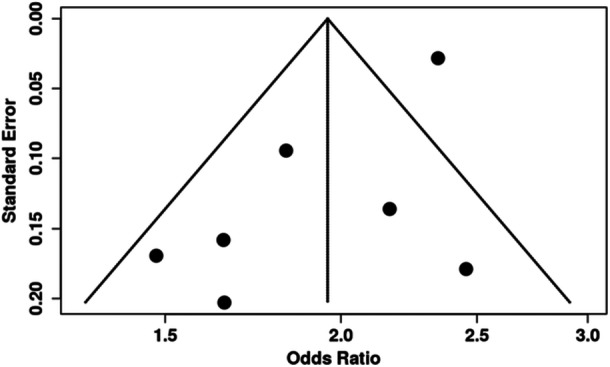
Funnel plot of the association between obstructive sleep apnea and periodontitis showing significant publication bias (*p* = 0.03).

There was a statistically significant association between OSA and periodontitis (7 studies: OR 1.96, 95% CI 1.68–2.29, *p* < 0.0001). However, significant heterogeneity (*I* ^2^ = 69.5%, *p* = 0.003) and publication bias (*p* = 0.039) were found. Applying the GRADE tool, the certainty of this outcome was rated as “low” (Table 2 and Figures 2 and 3).

To compensate for the publication bias, we conducted a trim‐and‐fill analysis. The pooled OR was higher than the original analysis (2.24, 95% CI 1.92, 2.60). This suggests that the “true” effect might be even stronger than what the published literature alone shows (Table 2 and Figure 2).

**Figure 4:**
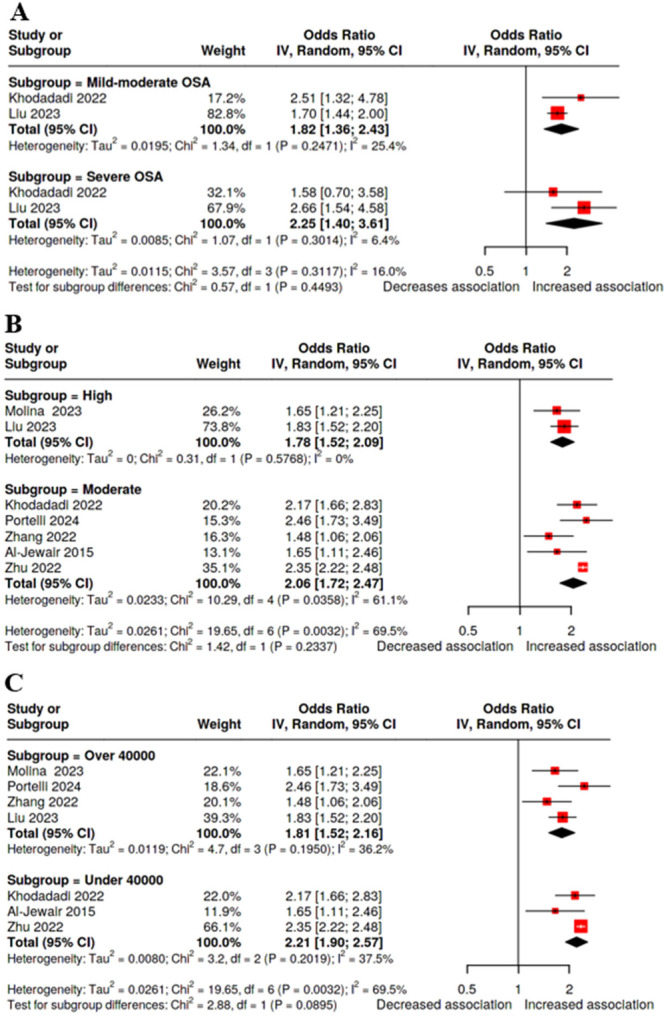
Sensitivity and subgroup analysis. A. According to the severity of obstructive sleep apnea. B. According to the quality of the studies. C. According to the sample size.

The pooled analysis of two studies showed some form of dose–response association between the variables, since the pooled OR for mild–moderate OSA was 1.82 versus 2.25 for severe OSA. However, the intergroup difference was not statistically significant (*p* = 0.44) (Table 2 and Figure 4).

Additionally, we conducted two subgroup analyses. Moderate‐quality studies showed a larger effect size than the high‐quality studies (OR 2.06 versus 1.78). Moreover, larger sample studies revealed a more modest effect size than the smaller size studies (OR 1.81 versus 2.21). However, none of these subgroup analyses reached statistical significance (Table 2 and Figure 4).

## 4 Discussion

This umbrella review synthesized evidence from seven MAs encompassing over 225,000 participants and demonstrated a significant association between OSA and periodontitis, with an overall pooled OR of 1.96 (95% CI: 1.68–2.29). The certainty of evidence for the main outcome was rated as low according to GRADE criteria, primarily due to concerns regarding heterogeneity and publication bias.

The overlap analysis revealed a very high overlap (26.8%) among the included MAs. This substantial overlap suggests evidence‐based saturation, where the same core studies are repeatedly synthesized rather than new primary research being conducted. The field would benefit more from new primary studies examining the OSA–periodontitis association rather than additional MAs. Thus, researchers should consider postponing new SRs until sufficient novel primary data emerges, and this high overlap may explain the downgrading of the evidence certainty in this umbrella review.

Sleep in general and OSA in particular have been studied extensively in relation to oral health, more specifically in the context of periodontitis. Sleep duration has emerged as a significant factor in periodontal health, with evidence suggesting that extremely short sleep duration (≤ 5 h) is associated with a 41% increased risk of periodontitis, though neither moderately short nor long sleep duration showed significant associations with periodontal disease (Zhou et al. 2024). Non‐apnea sleep disorders more broadly have demonstrated a 36% increased risk of severe periodontal disease, with particularly pronounced effects observed in younger and middle‐aged populations compared to individuals over 65 years (Lee et al. 2014). On the other hand, an SR and an MA of 13 studies demonstrated significant associations between periodontitis and three respiratory conditions, like chronic obstructive pulmonary disease (COPD), asthma, and pneumonia (Gomes‐Filho et al. 2020).

The relationship between edentulism and OSA has been consistently documented across 23 clinical studies published between 1999 and 2023, with tooth loss favoring anatomic alterations that deteriorate breathing and mean AHI scores in edentulous patients found to be 2–3 times higher compared to dentate individuals (Galtieri et al. 2024). However, the impact of complete denture use during sleep on OSA severity remains controversial, with an MA of four studies involving 144 patients showing no statistical difference in AHI between those wearing and not wearing dentures during sleep (Vila‐Nova et al. 2021).

Mandibular tori have been identified as potential predictors of OSA presence but not severity, with patients having mandibular tori showing a relative risk of 1.9 for OSA; hence, larger mandibular tori were associated with mild and moderate OSA rather than severe forms (Yong et al. 2025). Dental and orofacial structures are well‐established risk factors for OSA, but there is a wide range of knowledge about sleep apnea diagnosis, treatment, and referral from both general dentists and dental specialists. Polysomnography is recognized as the gold standard for diagnosing OSA by 40%–90% of practitioners who were surveyed (Alkharouby et al. 2025).

The oral microbiota of OSA patients differ significantly from control subjects; an overview of the results of eight studies, which included 344 OSA patients and 131 controls, showed that there was a significant difference in species and genera of bacteria related to periodontitis between these two groups, especially when the patients have mouth breathing (Bianchi et al. 2023). These microbiological changes may represent one mechanistic pathway connecting OSA to periodontal disease.

OSA has been proposed to influence dental caries risk through mechanisms involving reduced saliva flow and decreased oral self‐cleaning capacity, though this represents a related but distinct pathway from its effects on periodontal tissues (Lee et al. 2025). The chronic inflammatory burden and hyposalivation associated with OSA are suggested to affect periodontal status over time, though 10‐year follow‐up studies of hypertensive patients revealed that while OSA patients showed greater radiographic bone loss, no differences were observed in clinical periodontal parameters such as probing depth, bleeding on probing, or presence of inflammatory markers (Kvarnvik et al. 2024a).

Although obesity is a known risk factor for OSA, the data on OSA–periodontitis–obesity association is conflicting. For example, in a case–control study of 114 patients, periodontitis was 2 times higher in OSA than in non‐OSA patients, with BMI having an influential role (Pico‐Orozco et al. 2021). However, in morbidly obese Class III patients, the association between OSA risk and periodontal disease was not confirmed, with 81.5% showing high OSA risk and 97.2% presenting periodontal disease, yet no direct correlation between periodontal status and OSA risk emerged (Sales‐Peres et al. 2016). The complex interplay of multiple metabolic and inflammatory factors in severe obesity may obscure or modify the OSA–periodontitis relationship observed in general populations.

Comorbidities are important modifiers in the OSA–periodontitis association. In a case–control study of 60 participants, the most common conditions among people with OSA and periodontitis were found to be obesity and hypothyroidism. Severe OSA was found to be linked to both osteoarthritis and arterial hypertension (Arango Jimenez et al. 2023). A similar study found that women with hypertension or hypertensive cardiomyopathy were more likely to have OSA–periodontitis, while men with any of these two comorbidities were more likely to have severe OSA (Latorre et al. 2018). Risk for OSA among those who presented with both periodontitis and type II diabetes was found to be significantly higher than among those who had a diagnosis of periodontitis but no diabetes (82.5% versus 52.5%), indicating that the presence of two separate chronic inflammatory diseases may lead to an increased synergy in the susceptibility for developing OSA (Aishwaraya et al. 2024).

The continuous positive airway pressure (CPAP) is the standard treatment of OSA (Batool‐Anwar et al. 2016; Sutherland et al. 2014). Oral appliances (OATs) are an important alternative treatment for OSA, particularly effective for mild‐to‐moderate cases and especially due to a low level of patient compliance with CPAP use. An MA of 16 randomized controlled trials found that while CPAP was more effective than OATs at reducing OSA severity measures, OATs showed better patient preference and demonstrated particular benefits in severe OSA patients when using adjustable devices (Zhang et al. 2019).

We couldn't find any interventional study on whether regular periodontal therapy in the long term can decrease the severity of symptoms in established OSA cases. In a relatively relevant study, researchers compared OAT therapy plus a mouth shield with standard periodontal scaling and root planning. Both treatments produced similar periodontal improvements. The authors suggested this was likely because the OAT therapy reduced mouth breathing, which improved the oral environment on both sides of the mouth systemically (Lin et al. 2025). An SR found that while mandibular advancement appliance (MAA) use for OSA is associated with various oral side effects like hypersalivation and occlusal changes, there is insufficient evidence to determine whether MAA affects periodontal health due to limited research on this specific outcome (Mansour et al. 2024). In addition, based on the limited available evidence, CPAP does not appear to aggravate periodontitis (Incerti Parenti et al. 2025a; Kvarnvik et al. 2024).

Concerning the plausible biological mechanisms that connect OSA and periodontitis, the following links have been proposed in the literature: (1) Systemic inflammation and oxidative stress: Both diseases generate chronic oxidative stress and elevated inflammatory markers. OSA causes repeated cycles of hypoxia and reoxygenation during apneic episodes, producing reactive oxygen species that damage tissues systemically (Incerti Parenti et al. 2025). This oxidative burden can amplify periodontal inflammation and impair healing of gum tissues (Lavie et al. 2007). (2) Elevated inflammatory markers: Both OSA and periodontitis increase serum levels of inflammatory mediators, including C‐reactive protein, interleukin‐6, WBCs, and tumor necrosis factor‐α; these contribute to an environment favorable for inflammation‐related worsening of periodontitis (Corral et al. 2023; Tranfić Duplančić et al. 2022; Beydoun et al. 2020). (3) Altered oral environment: OSA patients are typically mouth breathers and experience reduced salivary flow at night due to upper‐airway obstruction, and since saliva provides beneficial antimicrobial activity as well as acts as a buffer against acid, it's reasonable that its absence would lead to proliferation of periodontal pathogens within periodontal pockets (Maniaci et al. 2024; Bokov et al. 2022). Moreover, OSA patients have been reported to have a significantly increased prevalence of *Porphyromonas gingivalis*, which is considered a keystone periodontal pathogen (Incerti Parenti et al. 2025). (4) Impaired endothelial function and decreased microcirculation: Both OSA and chronic periodontitis cause damage to the vascular endothelium (Kohler and Stradling 2010; Mercanoglu et al. 2004). Hypoxia caused by repeated periods of apnea during OSA causes microcirculatory problems in the gingiva. This could result in decreased oxygenation and nutrition to the periodontium and thereby decrease the capacity of the periodontal tissue against infection and to repair damaged periodontal tissues (Zeng et al. 2020). (5) Activation of the sympathetic nervous system (SNS): Each time an individual experiences an episode of apnea, a stress response occurs and the SNS is activated, resulting in increases in plasma cortisol and catecholamine. This may suppress both immune function and the process of wound healing, and this suppression could limit the body's ability to combat the infection present in the periodontium and repair damage to the periodontium (Arango Jimenez et al. 2023; Incerti Parenti et al. 2025). 6) Common underlying risk factors: Obesity, aging, smoking, alcohol, hypertension, and diabetes mellitus are among the recognized comorbidities for both OSA and periodontitis (Arango Jimenez et al. 2023; Incerti Parenti et al. 2025). These mechanisms probably act together and in a bidirectional manner so that each disease will exacerbate the other.

### 4.1 Strength and Limitations

The main strengths of this umbrella review are the large sample size encompassing over 225,000 participants, application of rigorous statistical techniques, and evaluation of the evidence certainty by the GRADE framework. However, it had some limitations also. The considerable heterogeneity among studies and publication bias limited the reliability and generalization of the results, eventually leading to a low level of certainty. In addition, the majority of included studies were cross‐sectional designs, limiting the ability to draw conclusions about causality.

### 4.2 Recommendations

Longitudinal studies are recommended, utilizing standardized diagnostic criteria to establish temporality and some form of causality. In order to create an integrated approach to address both diseases, interventional research is needed to find the impact of periodontal treatment on the severity of OSA and whether treatment of OSA patients with CPAP can affect periodontal outcomes.

## 5 Conclusions

The present umbrella review found a significant association between OSA and periodontitis. It seems that the association is generally higher in those with severe versus mild–moderate OSA. However, as per GRADE criteria, the level of confidence in this association is currently low due to the observational nature of the studies and methodological limitations. Overall, the study results support the implementation of an integrative approach to patient care, where OSA patients are regularly monitored for their periodontal health status. Longitudinal studies employing standardized diagnostic criteria would determine if treatment of one disease has an effect on the outcome of the second.

## Author Contributions

**Mohammed Khalid Mahmood:** conceptualization, methodology, data curation, formal analysis, investigation, writing – original draft, writing – review and editing. **Shahen Hiwa Omer:** methodology, validation, writing – original draft, writing – review and editing. **Sarhang Sarwat Gul:** data curation, formal analysis, writing – original draft, writing – review and editing. **Yad Mariwan Mohammed Amin:** validation, investigation, writing – original draft, writing – review and editing. **Herve Tassery:** supervision, resources, writing – original draft, writing – review and editing. **Delphine Tardivo:** supervision, validation, writing – original draft, writing – review and editing. **Romain Lan:** conceptualization, supervision, writing – original draft, writing – review and editing.

## Funding

The authors received no specific funding for this work.

## Ethics Statement

The authors have nothing to report.

## Consent

The authors have nothing to report.

## Conflicts of Interest

The authors declare no conflicts of interest.

## Supporting information


**Supplementary Table 1:** Search strategy of the umbrella review.
**Supplementary Table 2:** Methodological quality assessment of the included systematic reviews using AMSTAR 2.
**Supplementary Table 3:** Overlap analysis of the included studies.

## Data Availability

Data supporting the content of this article are present in the Supplementary Material.
