# Supplementary material for: Association Between Obstructive Sleep Apnea and Periodontitis: A Systematic Umbrella Review
Source: Clin Exp Dent Res. 2026 Apr 26;12(2):e70300. doi: 10.1002/cre2.70300 (PMC13110838; doi:10.1002/cre2.70300)
Supplement: Supplementary file 1 — Supplementary Table 1: Search strategy of the umbrella review. Supplementary Table 2: Methodological quality assessment of the included systematic reviews using AMSTAR 2. Supplementary Table 3: Overlap analysis of the included studies. [file CRE2-12-e70300-s001.docx]

**Supplementary material for the article:**

**Association between periodontitis and obstructive sleep apnea: a systematic umbrella review**

**Supplementary Table 1. Search strategy of the umbrella review**

| **Databases** | **Search strategy** |
| --- | --- |
| **PubMed/MEDLINE** | (  ("Periodontitis"[Mesh] OR "Periodontal Diseases"[Mesh]  OR periodontitis[tiab] OR periodontal disease*[tiab]  OR periodontal[tiab] OR periodont*[tiab]  OR gingivitis[tiab] OR "Gingivitis"[Mesh])  )  AND  (  ("Sleep Apnea, Obstructive"[Mesh] OR "Sleep Apnea Syndromes"[Mesh]  OR obstructive sleep apnea[tiab] OR obstructive sleep apnoea[tiab]  OR sleep apnea[tiab] OR sleep apnoea[tiab]  OR sleep-disordered breathing[tiab] OR OSA[tiab] OR SDB[tiab])  )  AND  (  "systematic review"[pt] OR "meta-analysis"[pt]  OR "systematic review"[tiab] OR "systematic reviews"[tiab]  OR "meta-analysis"[tiab] OR "meta-analyses"[tiab]  OR "umbrella review"[tiab] OR "overview of reviews"[tiab]  OR "review of reviews"[tiab]  )  AND english[lang] |
| **Web of Science** | TS = (  periodontitis OR periodontal disease* OR periodontal OR gingivitis  )  AND  TS = (  "obstructive sleep apnea" OR "obstructive sleep apnoea"  OR "sleep apnea" OR "sleep apnoea"  OR "sleep-disordered breathing" OR OSA OR SDB  )  AND  TS = (  "systematic review" OR "systematic reviews"  OR "meta-analysis" OR "meta-analyses"  OR "umbrella review" OR "overview of reviews"  OR "review of reviews"  )  AND LA = (English) |
| **Embase** | (  'periodontitis'/exp  OR 'periodontal disease'/exp  OR periodontitis:ti,ab  OR periodontal:ti,ab  OR 'periodontal disease*':ti,ab  OR gingivitis:ti,ab  )  AND  (  'obstructive sleep apnea'/exp  OR 'sleep disordered breathing'/exp  OR 'sleep apnea'/exp  OR 'obstructive sleep apnea':ti,ab  OR 'obstructive sleep apnoea':ti,ab  OR osa:ti,ab  OR 'sleep-disordered breathing':ti,ab  OR sdb:ti,ab  )  AND  (  'systematic review'/exp  OR 'meta analysis'/exp  OR 'umbrella review'/exp  OR 'systematic review':ti,ab  OR 'meta-analysis':ti,ab  OR 'umbrella review':ti,ab  OR 'overview of reviews':ti,ab  OR 'review of reviews':ti,ab  )  AND  [english]/lim |
| **Google Scholar** | allintitle: periodontitis OR "periodontal disease" "obstructive sleep apnea" OR OSA "systematic review" OR "meta-analysis" |

**Supplementary Table 2. Methodological quality assessment of the included systematic reviews using AMSTAR 2**

| **Author, year [reference]** | **Q1** | **Q2**  **(C)** | **Q3** | **Q4**  **(C)** | **Q5** | **Q6** | **Q7**  **(C)** | **Q8** | **Q9**  **(C)** | **Q10** | **Q11**  **(C)** | **Q12** | **Q13**  **(C)** | **Q14** | **Q15**  **(C)** | **Q16** | **Quality** |  |
| --- | --- | --- | --- | --- | --- | --- | --- | --- | --- | --- | --- | --- | --- | --- | --- | --- | --- | --- |
| Al-Jewair et al. 2015 | Y | Y | Y | Y | Y | Y | Y | Y | Y | N | Y | PY | PY | PY | N | Y | Moderate |  |
| Khodadadi et al. 2022 | Y | N | PY | Y | Y | Y | N | Y | Y | N | Y | N | PY | Y | Y | Y | Moderate |  |
| Molina et al. 2023 | Y | Y | PY | Y | Y | PY | Y | Y | Y | Y | Y | PY | Y | Y | Y | Y | High |  |
| Portelli et al. 2024 | Y | Y | PY | Y | Y | PY | N | Y | Y | N | Y | N | PY | Y | Y | Y | Moderate |  |
| Liu et al. 2023 | Y | N | Y | Y | Y | Y | N | Y | Y | N | Y | Y | Y | Y | Y | Y | High |  |
| Zhang et al. 2022 | Y | Y | PY | Y | Y | Y | N | Y | Y | N | Y | N | N | Y | Y | Y | Moderate |  |
| Zhu et al. 2022 | Y | N | Y | Y | Y | Y | N | Y | Y | N | Y | Y | Y | Y | N | Y | Moderate |  |
| **Ref: references, C: critical domain, N: No, NA: not applicable, PY: partially yes, Y: Yes. Q1: Did the research questions and inclusion criteria for the review include the components of PICO?, Q2: Did the report of the review contain an explicit statement that the review methods were established prior to the conduct of the review and did the report justify any significant deviations from the protocol?; Q3, Did the review authors explain their selection of the study designs for inclusion in the review?; Q4, Did the review authors use a comprehensive literature search strategy?; Q5 Did the review authors perform study selection in duplicate?; Q6, Did the review authors perform data extraction in duplicate?; Q7, Did the review authors provide a list of excluded studies and justify the exclusions?; Q8, Did the review authors describe the included studies in adequate detail?; Q9, Did the review authors use a satisfactory technique for assessing the risk of bias?; Q10, Did the review authors report on the sources of funding?; Q11, Did the review authors use appropriate methods for statistical combination of results?; Q12, Did the review authors assess the potential impact of RoB in individual studies on the results?; Q13, Did the review authors** **account for RoB in individual studies when interpreting/ discussing the results of the review?; Q14, Did the review authors provide a satisfactory explanation for, and discussion of, any heterogeneity?; Q15, Did the review authors carry out an adequate investigation of publication bias?; Q16, Did the review authors report any potential sources of conflict of interest?** | | | | | | | | | | | | | | | | | | |

**Supplementary Table 3. Overlap analysis of the included studies**

| **Metric** | **Value** |
| --- | --- |
| Number of meta-analyses | 7 |
| Total unique studies | 23 |
| Total study inclusions | 60 |
| Studies in >1 meta-analysis | 15 (65.2%) |
| **Corrected Covered Area (CCA)** | **26.8%** |
